# Bioactive Compounds from Agricultural Residues, Their Obtaining Techniques, and the Antimicrobial Effect as Postharvest Additives

**DOI:** 10.1155/2021/9936722

**Published:** 2021-09-17

**Authors:** Rafael Gomes-Araújo, Dolores Gabriela Martínez-Vázquez, Ana Verónica Charles-Rodríguez, Sarahi Rangel-Ortega, Armando Robledo-Olivo

**Affiliations:** Bioprocess Agrofood Research Group, Food Science and Technology Department, Universidad Autónoma Agraria Antonio Narro, Saltillo 25315, Mexico

## Abstract

Agricultural vegetable products always seek to meet the growing demands of the population; however, today, there are great losses in supply chains and in the sales stage. Looking for a longer shelf life of fruits and vegetables, postharvest technologies have been developed that allow an adequate transfer from the field to the point of sale and a longer shelf life. One of the most attractive methods to improve quality and nutritional content and extend shelf life of fruits and vegetables is the incorporation of bioactive compounds with postharvest technologies. These compounds are substances that can prevent food spoilage and the proliferation of harmful microorganisms and, in some cases, act as a dietary supplement or provide health benefits. This review presents an updated overview of the knowledge about bioactive compounds derived from plant residues, the techniques most used for obtaining them, their incorporation in edible films and coatings, and the methods of microbial inhibition.

## 1. Introduction

At present, agricultural problems are based on the adequate food production to satisfy global consumers. However, such production must focus on sustainable processes, thus avoiding generating pollution and waste derived from the agricultural process. It is useless to implement intensive and organic production technologies, if there are postharvest losses within the supply chain during transport, at the sales stage, or during the storage process. Much of the food waste occurs during the postharvest stage, when the product of interest is removed from the soil or separated from the mother plant and its deterioration begins. The routes of products from the countryside to urban areas, complex marketing systems, and the international trade are the main points of attention for the implementation of postharvest techniques, in order to preserve the quality of the products for as long as possible. The plant tissues when exposed to air, accompanied with changes in temperature, solar radiation, and some cuts or blows, begin a process of physiological deterioration. Some crops are even affected at the preharvest stage, where the limitation of nutrients or the impact of some conditions of the crop in its final phase limits the shelf life of the product in its postharvest stage [1].

The global agenda established by the United Nations towards 2030 has determined various objectives for sustainable development. In its objective number 12-Responsible Consumption and Production, it seeks a joint effort among nations to reduce food loss and the generation of food waste. Food losses are quantified from production to the retail level, while food waste comprises the retail and consumption levels [2]. In a study carried out by FAO et al. [2] during 2016, the percentage of food losses by region was analyzed, generating an average global loss of 13.80%, representing losses above 400 billion USD per year.

The significance of postharvest processes does not only lie in the conservation of agricultural products but also in the nutritional quality provided by having food in good condition or even with the addition of beneficial compounds for health. As is well known, fresh fruits and vegetables are a source of vitamins and minerals that can benefit human health. Postharvest deterioration decreases the benevolent capacity of these compounds, in addition to the generation of substances harmful to health.

Due to the importance of the application of postharvest technologies, the obtaining of quality food and enough quantity arises. Postharvest technologies are mainly based on controlling the storage environment or handling conditions, including temperature and humidity. Bioactive compounds are substances that can interfere with cell senescence; the reason why is they have been incorporated into postharvest technologies allowing to extend the shelf life of storage and transport stages. These bioactive compounds are generally found in the same foods or plants from where they are extracted by various techniques [3]. Bioactive compounds mainly exhibit antioxidant and antimicrobial properties [4], which make them attractive for incorporation into minimally processed and horticultural product preservation technologies.

## 2. Main Bioactive Compounds Present in Plant Waste

The circular economy is a system for the use of resources where the reduction of elements prevails, which applies different mechanisms with the aim of minimizing the generation of waste, thus releasing the economic growth of natural resources. Every year, it is estimated that a third of all food produced is wasted [5], whereof 20.4 million tons is wasted in Mexico [6]. In the fruit and vegetable sector, 45% of the total produced is lost in the production chains (postharvest, processing, and distribution) and consumption [7, 8]. Currently, world postharvest losses of fruits and vegetables caused by microorganisms are of the order of 5 to 25% in developed countries and between 20 and 50% in developing countries. This waste represents the origin of contamination of the soil, air, and water sources, affecting both the environment and public health [9].

In Mexico, the fruit processing industries (lemon, pears, tomato, apple, papaya, pineapple, banana, and oranges), cereals (corn), and vegetables (beans, cabbage, carrots, lettuce, and potatoes) generate around 76 million tons of annual waste [10]. Therefore, it is necessary to find new technological and environmentally friendly solutions, to use fruit and vegetable waste as new raw materials, in order to develop and expand the production of bioproducts with high added value.

Agroindustrial waste has a great potential to generate food additives that can be beneficial to ensure global food sustainability [11]. Considering their high removal rates, by-products of plant sources can be used to obtain commercially valuable bioactive products [12]. A bioactive compound is a substance that has biological activity. In a broader sense, it is a substance that has an effect or can trigger a physiological response in a living organism. As they come from plant sources, bioactive compounds are phytoconstituents that are part of the food chain and are responsible for numerous beneficial changes in health. For example, antioxidant, anticancer, anti-inflammatory, antiallergic, antiatherogenic, and antiproliferative properties are the main bioactivities of the bioactive compounds [13].

Bioactive compounds are synthesized from some plant primary metabolites (e.g., amino acids) or from intermediate compounds obtained by primary metabolism in specialized cell types only during a particular growth stage, or under specific conditions, making their extraction and purification difficult [14]. Commercially useful bioactive compounds (secondary metabolites) are terpenoids, polyphenols, vitamins, alkaloids, etc. [15], which have been used to prevent the risk of diseases and in the treatment of a wide range of illness. In plants, they have a protective role against biotic and abiotic stress [16]. Given that bioactive compounds are present in different amounts, it is important to develop their production, in order to obtain as much as possible, and find new sources cheaper and alternative [17, 18]. Therefore, the materials considered waste can be a valuable source of bioactive compounds, able to be extracted and implemented in new processes and products for the replacement of chemical food additives.

The by-products of the fruit processing industry such as bark, peel, seeds, and pomace represent the largest amount of waste, which is made up of a wide variety of bioactive compounds with multiple biological properties. The exploitation of residues for the development of food products with added value could allow the generation of additional benefits. It is currently known that the cost of technologies for the purification of bioactive compounds exceeds the cost of reprocessing, so that the full use of waste with functional properties as additives could lead the food industry to reduce waste and increase its profitability [14].

Fruit and vegetable waste is mainly composed of lignocellulosic biomass, which is made up of cellulose (30–50%), hemicellulose (15–35%), and lignin (10–20%) [19], being a viable source of sugars and phenolic compounds. Phenolic compounds are classified as primary antioxidants that are free radical scavengers that inhibit lipid oxidation, reducing the formation of volatile compounds (aldehydes and ketones) that cause rancidity [20]. Phenolic compounds are important for their antioxidant properties and their protection against degenerative diseases such as cancer and heart disease [21]. Phenolic acids are a group of derivatives of benzoic and cinnamic acids such as capsaicin, ellagic, salicylic, tannic, vanillin, gallic, syringic, p-coumaric, o-coumaric, m-coumaric, caffeic, ferulic, sinapinic, and chlorogenic acids occurring in both forms, free and bound [9]. Flavonoids are compounds that consist of two aromatic rings joined by a three-carbon bond. Furthermore, flavonoids belong to different subclasses such as anthocyanins, flavonols, flavanones, flavones, and isoflavones. The structure of stilbenes is represented by two phenyl rings linked together by a bridge of two carbon atoms such as resveratrol and viniferine [20]. On the other hand, lignans are a complex, heterogeneous polymer, formed mainly by phenylpropanoid derivatives that correspond to the so-called monolignols (p-coumarilic, coniferyl, and sinapyllic alcohols) [16]. Coumarins are also simple phenolic compounds that have their chain side forming a cyclic structure (lactones or phenylpropanoids). Finally, tannins are grouped into two main classes: hydrolysable tannins, which are characterized by having a glycoside structure, and condensed tannins, which are polymers whose structures are related to flavonoid compounds (also called proanthocyanins, proanthocyanidins, or leucoanthocyanidins).  Table 1 presents the main bioactive compounds in various plant residues with antimicrobial, anti-inflammatory, anticancer, antiallergic, antithrombotic, cardioprotective, and vasodilator activities.

## 3. Overview of the Methods for Obtaining Bioactive Compounds Derived from Plant Residues

The discovery of the extraction of bioactive compounds and their applications as active principles for the treatment of diseases has led to an accentuated development of human society. Natural bioactive compounds play an important role in daily activities, associated with many health benefits and low toxicity or negative effects. In recent years, a high demand for natural bioactive compounds has been generated, creating the need to develop suitable methods, equipment, or strategies for the extraction of such compounds from natural sources [50–52].

The selection of the extraction method and the operational parameters of the process is always based on the overall performance of bioactive compounds. However, it is necessary to determine the family of compounds or the specific compound to be extracted, in order to optimize the operating conditions [51].

The strategy for the extraction of bioactive compounds from agroindustrial waste should aim to maximize the removal performance of bioactive compounds and create the minimum residue. The selected method should be adapted to satisfy the demands of industrial processing, such as bioactive compound purity and its conservation conditions to avoid deterioration and oxidation of the compounds, as well as ensure the quality characteristics of the final product and the sustainability of the process [53].

The bioactive compounds from agroindustrial residues such as fruits, vegetables, and plants can be extracted by different methodologies, which are described below. The extraction processes can be influenced by many factors including the solvent and matrix properties, particle size, process pressure, time, temperature, and the solvent-matrix ratio [54].

### 3.1. Solid-Liquid Extraction

The solid-liquid extraction, commonly called maceration, is the oldest technique to obtain nonvolatile compounds from plants, by using different solvents in contact with the vegetal matrix. This removal technique is the basis for many other new technologies to enhance extraction yields of bioactive molecules and reduce the time of separation, inducing to solvent and energy saving [53–55]. The yield of a solid-liquid extraction depends on the type of the polarities of the solvents, the mixture pH, the extraction time, and temperature, as well as on the chemical composition of the sample and the particle size [51].

The new technologies are based in solid-liquid extraction and are named “assisted” technologies such as microwave, ultrasonic, pressure, electrical technologies (ohmic heating), enzymatic, and mechanical (pressurized hot water extraction and subcritical fluid extraction) treatments. These new technologies are suitable for improving the recovery of valuable compounds, with different properties and bioactivities, since they use green solvents and allow plant cell disruption with better compound extraction, while minimize the impact on bioactive compounds [55].

### 3.2. Microwave-Assisted Extraction

Microwave-assisted extraction (MAE) is a solid-liquid extraction with microwave heating. The microwaves are electromagnetic waves whose frequency varies between 300 MHz and 300 GHz. The microwave heating is produced by the capacity of the samples to absorb the microwave energy and convert it into heat. The conversion of microwave energy into heat occurs due to dipolar and ionic mechanisms. The presence of polar molecules is the cause of microwave heating due the dipolar nature of water. Microwave generates an oscillating electric field, and the permanently polarized dipolar molecules try to realign in the direction of the electric field, causing internal friction of the molecules resulting in the heating of the material [56–58].

The principal advantages of MAE are the high yields of extraction, compatibility with the environment, short extraction time, rapid temperature increase, high efficiency, better monitoring of the process, and low energy consumption and cost [59]. MAE efficiency for bioactive compounds extraction depends on particle size, irradiation time, power, and solid-to-liquid ratio. MAE has been applied to extract many natural compounds from agroindustrial residues from vegetables and fruits to obtain bioactive compounds, mainly antioxidants, polysaccharides, and oils rich in carotenoids and polyphenolic compounds [60].

### 3.3. Ultrasonic-Assisted Extraction

Ultrasonic-assisted extraction is a widely used technology to extract bioactive compounds from many agroindustrial wastes. The principle is based on the effects of cavitation, which intensify the mass transfer between the solvent and the matrix. The collapse of cavitation bubbles near tissue surfaces produces microjet impingement, resulting in tissue disruption and deep penetration of the solvent into the tissue matrix, increasing the extraction yield. Ultrasonic extraction offers an advantage in terms of shorter processing time and enhanced quality, and it is considered an environmentally friendly technology [46, 61].

### 3.4. Subcritical Water Extraction

Extraction with subcritical water is a recent method to extract bioactive compounds that uses solvents under high temperature (100-374°C) and critical pressure (1–22.1 MPa), to dissolve active substances from the raw materials. Due to these extreme conditions, the solubility of the compounds of interest is increased, making it possible to use water instead of organic solvents. The addition of ethanol helps reducing the extraction temperature and the concentration of toxic compounds, generated by the Maillard reaction in agroindustrial waste. Subcritical extraction has the advantages of obtaining high extraction yields, with an extremely low number of solvents and short operating times. However, there are some disadvantages: the need to raise the temperature, and for the extraction of several heat-sensitive compounds, high temperatures are not suitable and they result degraded [51, 62, 63].

### 3.5. Supercritical Fluid Extraction

Supercritical fluid extraction is a green extraction method that has been widely used to obtain bioactive compounds from different raw materials, with the application of carbon dioxide as a renewable and green solvent. The low price of extraction, low temperature of extraction, and no toxicity of carbon dioxide make this method of great option to extract bioactive compounds from agroindustrial residues [64, 65].

### 3.6. Membrane Technology

The final disposal of agroindustrial aqueous waste has become a major challenge for food processing industries, due to its negative impact on the environment. Pressure-driven membrane processes such as micro-, nano-, and ultrafiltration have several benefits in the treatment of wastewaters: high separation efficiency, low energy requirements, easy scale-up, simple operation, high productivity, and the absence of phase transition. Many bioactive compounds have been recovered from agroindustrial wastes using membrane technology, such as antioxidant compounds, carbohydrates, pectin, proteins, sugars, and phenolic compounds [66].

### 3.7. Fermentation and Enzymatic Process

The production or biotransformation of bioactive compounds through fermentation or enzymatic processes from agroindustrial residues has increased in recent years. Solid-state fermentation (SSF) is a bioprocess where the enzymes released by a microorganism (bacteria, fungi, or yeast) act as biocatalysts and allow better release and extraction of bioactive compounds from different substrates. SSF is an economical biotechnological process, easy to implement as it requires small equipment and low capital investment. The enzymatic processes are more complex, are expensive, and have some limitations such as low stability, recovery, and temperature sensitivity that can be reduced using enzyme immobilization [55]. Most microorganisms used are filamentous fungi such as *Trichoderma* and *Aspergillus*, due to its high production of secondary metabolites, and enzymes that can be used to metabolize agroindustrial residues to produce different bioactive compounds [67].

## 4. Edible Films and Coatings as Postharvest Techniques

To avoid or reduce the adverse postharvest effects, different technologies have been implemented. Between the most used methods are storage at low temperatures, the application of gamma and ultraviolet radiation, conservation in controlled atmosphere, and use of plastic packaging, among others [68, 69]. Seeking for the improvement in the nutritional quality and commercial value of horticultural products, there is research focused on the development of edible films and coatings. They provide the possibility of enhancing the safety of the product by acting as a barrier against the transport of water vapor, oxygen, and compounds responsible for flavor, color, and aroma [70–73].

An edible film (EF) is a preformed matrix, obtained by molding, while an edible coating (EC) is a transparent, continuous, edible, and thin matrix, which is placed on the surface of the food by immersing or spraying the coating-forming solution, whose thickness is always less than that of EFs [74]. Both the EF and EC have the purpose of preserving the quality of the product, serving as packaging, and extending its useful life.

**Figure 1:**
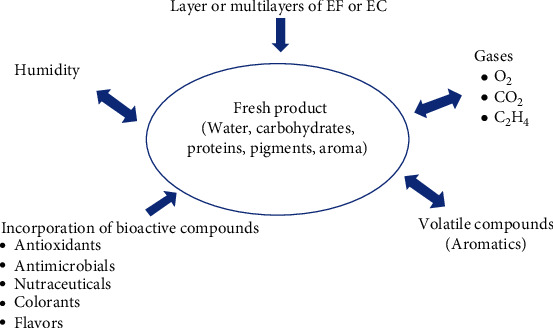
Barrier and transport effect of an edible coating (EC) and/or an edible film (EF) (adapted from Falguera et al. [76]).

The EF and EC forming solutions are setup of components that are divided into three main categories, as indicated in Table 2. However, the formulation of both the films and the coatings will depend on the function that they will carry out. Additionally, both can act as supports that contain bioactive compounds such as antimicrobials, antioxidants, pigments, and compounds that provide flavor and aroma, preservatives, vitamins, and minerals, among others (Figure 1). Within the compounds that are usually used to provide stability to EF and EC are emulsifiers, surfactants, and plasticizers. The emulsifier holds the components together in the forming solution; the surfactant reduces the surface tension of the formulation, achieving greater uniformity; the plasticizer modifies its mechanical properties [75].

The use of edible films and coatings has proven to be an effective method of food preservation. However, when coating a fruit or vegetable, it is necessary that there should be a certain permeability to oxygen and carbon dioxide, in order to avoid anaerobic respiration. This anaerobic respiration could induce physiological disorders and a rapid loss of the quality and shelf life in food [77]. For this reason, the proper selection of the formulation is of utmost importance.  Table 3 shows various studies of the components used as EC and/or EF, as well as their effects on fruit and vegetable products.

## 5. Microbial Inhibition Methods by Bioactive Compounds When Incorporated in the Postharvest Stage

The postharvest decomposition of fruits and vegetables by the action of pathogenic microorganisms represents significant levels in the losses of these products [99]. The incorporation of bioactive compounds in the postharvest of fruit and vegetable products has resulted in controlling the incidence of pathogenic microorganisms [100].  Table 4 shows some examples of antimicrobial activity of bioactive compounds recovered from plant residues and applied on plant products in the postharvest stage.

The exploration of natural plant products as a source of bioactive compounds for the protection of postharvest loss of fruits and vegetables is in its early stages. The information available on the mechanisms of action of plant extracts is scarce; its antimicrobial activity is probably attributable to more than one mode of action [101]. However, there are different sources that have cited the mechanisms by which these compounds exert their antimicrobial effects, mainly inhibiting deteriorating fungi and pathogenic bacteria.

Anibal et al. [102] reported that the antifungal effects of extracts of the pericarp and pomegranate peel with high amounts of punicalagin and tannins are attributed to changes in the structure and cell morphology of the *Candida* genus. When observed by electron microscopy, the treated cells presented irregular cell walls, with viscous material on the surface as well as hyphal rupture and desquamation.

It has also been described that aldehyde, a group of volatile compounds, interferes in fungal cell division by reacting and inactivating sulfhydryl, a functional group involved in the division of fungal cells. Compounds such as cinnamaldehyde (extracted from cinnamon), citral (extracted from lemon grass), and perillaldehyde (extracted from the perilla plant) are good electron acceptors. These compounds act by disrupting fungal metabolism by forming a charge transfer complex with electron donors present in fungal cells [103].

Besides, the polyphenols of green tea (*Camelia sinensis*) have been studied for their antifungal capacity against the fungus that affects rice. The study showed a change in the permeability of the membrane of the rice fungus when presenting an increase in the percentage of electrolyte leakage. Since phenolic hydroxyl groups can bind the hydrophilic end of the lipid bilayer to agglomerate the lipid of the membrane, thus, these damage the cell membrane and promote electrolyte leakage. Furthermore, green tea polyphenols have shown antimicrobial activity at high doses of epigallocatechin gallate (EGCG), damaging the liposome membrane in *E. coli* and *S. aureus*, leaking intramembrane materials and consequently the aggregation of liposomes. In addition, these polyphenols have been shown to strongly inhibit the biofilm formation of pathogenic *E. coli* strains, by reducing the expression of the regulatory protein “CsgD,” a crucial activator in cellulose biosynthesis [104].

The antimicrobial capacity of some phenolic compounds extracted from mango residues, such as galangin, is attributed to the inhibition of cellular DNA gyrase in bacteria. The inhibition of the enzyme topoisomerase IV, as well as the anti-beta-lactamase activity (bind metals and proteins, affecting their bioavailability), generates nonspecific effects that trigger antioxidant and antimicrobial phenomena [105].

Organic acids have presented a mechanism of action in the inhibition of microorganisms that are related to acid-base balance, proton donation, and cell energy production. The cell of microorganisms in a normal state maintains equilibrium by establishing an internal pH close to neutrality. Cell homeostasis was defined as the establishment of a chemical balance in the face of environmental changes, damage to microbial cells, causing their alteration. When a change in pH occurs, proteins, nucleic acids, and phospholipids of the microbial cell can be structurally altered [106].

Various publications suggest that the main mechanism of the antimicrobial effects of volatile compounds extracted from different fruits indicates potential damage to the bacterial membrane, generating changes in permeability, polarization, and interruption of flow activity. Phenolic compounds have shown the ability to modify the regulation of genes associated with certain virulence attributes in bacteria, including hydrophobicity, adherence, motility, invasion, and biofilm generation. The above indicates the potential of phenolic compounds extracted from fruits as antimicrobials [107].

Other authors have described the mechanisms of action that some bioactive compounds generally present. Lauzardo et al. [108] mention that the toxicity effect of phenols on microorganisms can be attributed to enzymatic inhibition of compound oxidation. Terpenes and essential oils, although not fully studied, can cause membrane rupture by lipophilic compounds. The alkaloids are embedded in the DNA, while lecithins and polypeptides form ion channels in the cell membranes of microorganisms or, by competitive inhibition, cause adhesion of microbial proteins to the host's receptor polysaccharides.

## 6. Conclusions

Bioactive compounds are lined up to help revolutionize postharvest technologies and achieve improved increases in the shelf life of fruits and vegetables. The different protection mechanisms they provide make them versatile to the various forms of application of current postharvest technologies. Phenolic compounds and their derivatives are to date the substances with the greatest protection properties against oxidative stress, as well as against the attack of microorganisms.

The use of residues of plant origin follows the ideology of circular bioeconomy, seeking sustainable processes that allow to earn an added value. Plant-based waste is a rich source for obtaining bioactive compounds. The challenge lies in the improvement of the current technologies or looking for new ones that allow a higher percentage of obtaining bioactive compounds, as well as methods for incorporation into fruit and horticultural products. Further research should be made focused on the exploration of novel sources of bioactive compounds from vegetal wastes, their green extraction methods, and the evaluation of microbial inhibition mechanisms. The literature indicates that the main mechanism of action for the compounds extracted from plant sources as antimicrobial agents is damage to the cellular structure. Although there is information that allows to explain these effects, more studies are necessary for a complete understanding of the different mechanisms of action by bioactive compounds on the presence of spoilage and pathogenic microorganisms in the postharvest stages.

## Figures and Tables

**Table 1:** Major bioactive compounds present in various plant residues.

Source	Residue	Bioactive compound	Reference
Fruits			
Citrus	Peel	Limonene, *γ*-terpinene	[22]
		Polyphenols, carotenoids, limonoids	[23]
Grape	Seed	Catechin, epicatechin gallate, flavonoids	[24]
		Anthocyanins, phenolic compounds	[25]
	Peel	Phenolic compounds	[26]
		Anthocyanins	[27]
		Flavonoids	[28]
Pineapple	Husk	Phenolic compounds	[29]
Acerola	Waste	Flavonoids and anthocyanins	[30]
Papaya	Seeds	Sulforaphane and phenolic compounds	[31]
Avocado	Seeds, husk, and damaged pulp	Phenolic compounds, acetogenic, carotenoids	[32]
Strawberry and raspberry		Phenols, sugars, uronic acids, and anthocyanins	[33]
Apple	Pomace	Flavonoids, hydroxycinnamic acid, dihydroxy alkaloids	[34]
	Peel	Phenolic compounds	[35]
Kiwi	Peel, seed	Gallic acid, catechin, rutin, quercetin, ferulic acid, vanillin	[36]
Orange and mango	Peel	Carotenoids, limonene, ascorbic acid, flavonoids, phenylpropanoids	[37]
Purple eggplant	Peel and pulp	Anthocyanins and phenolic compounds	[38, 39]
Jabuticaba	Peel	Anthocyanins and phenolic compounds	[40]
Pomegranate	Husk	Phenolic compounds	[41]
Passion fruit	Rinds and bagasse	Scirpusin B, piceatannol	[42]
Vegetables			
Cardamom, radish, turnip	Aerial part and leaves	Phenolic compounds	[43]
Opuntia spp.		Phenols, betalain	[44]
Lettuce	Waste	Antioxidant extracts	[45]
Tobacco	Tobacco industrial waste	Alkaloids, phenolic compounds, terpenes, and terpenoids	[46]
Carrot	Pulp	Phenolic compounds	[35]
Tomatoes	Waste	Sterols, tocopherols, carotenes, terpenes, and polyphenols	[47]
Beet	Leaves and stems	2-Ethyl-1-hexanol, 2,2-dimethoxy-1,2-diphenyl-ethanone, 1,1,3-trimethoxypropane	[48]
Red cabbage Brussels sprout waste	Sprout residue	Phenolic acids, glycosylated flavonoids, acetylated flavonoids, anthocyanins	[49]

**Table 2:** The main components of edible films and coatings (adapted from Espitia et al. [75]).

Components		Characteristics
Hydrocolloids	Proteins, such as casein, gelatin, wheat gluten, corn protein, soy, and whey	High permeability to water vapor, barrier to oxygen and carbon dioxide, mechanical strength, can be soluble or insoluble in water
	Polysaccharides, such as starch, alginate, pectin, cellulose derivatives, chitosan, dextrin, carrageenan, and acacia	
Lipids	Waxes, fatty acids, acetylated monoglycerides, sucrose fatty acid esters, and shellac	High oxygen permeability, water vapor barrier, provide gloss, low structural strength, and durability
Mixtures	EF of casein and acetylated monoglycerides	Combination of hydrocolloid and lipid properties

**Table 3:** Edible coatings and/or edible films that are applied to fruits and vegetables to prolong their postharvest quality.

Fruit and vegetable product	Formulation	Effect	Reference
Pear	Methylcellulose	Browning reduction	[78]
Strawberry	Cactus mucilage	Permanence of texture, color, and sensory attributes	[74]
Mango	Chitosan	Reduction in water loss, sensory properties, and inhibition of the growth of microorganisms	[79]
Apple	Alginate, gellan gum	Reduction of moisture loss, slowing down of respiration	[80]
Banana	Ascorbic acid + calcium chloride, cysteine + carrageenan	Reduction of enzymatic browning and maintenance of firmness	[81]
Strawberry	Flaxseed mucilage + chitosan	Barrier to gases, reduction of moisture loss, and antifungal effect	[72]
Broccoli	Chitosan	Reduction of mesophilic microbial load	[82]
Banana	Chitosan + Arabic gum	Control of the fungus that causes anthracnosis in the fruit (*Colletotrichum musae*)	[83]
Grape	Chitosan + carboxymethylcellulose	Decreased respiratory rate and increased mechanical resistance	[84]
Mango	Potato and cassava starch	Preservation of appearance, color, firmness, and reduction of respiration	[85]
Tomatoes	Cassava starch	Effect on the ripening process	[86]
Strawberry	Chitosan + starch with cinnamon essential oil	Preservation of total phenol content and antioxidant capacity, delay of microbial development	[87]
Mango	Chitosan + lemon and orange essential oils	Reduction of coliforms, psychrophiles, fungi, and yeasts	[88]
Pepper	Biopolymers (gums : Arabic, xanthan, and pectin) + candelilla wax + jojoba oil + tar extract	Permanence of quality physicochemical parameters, as well as an increase in shelf life	[89]
Carrot	Beeswax + gums (guar and xanthan) + canola oil + propolis tincture	Inhibition of mold and yeast growth; reduction of weight loss and color	[90]
Tomatoes	Bay wax + olive oil + Tween 80 + propylene glycol + glycerol + glucose	Good functional and mechanical characteristics; reduced weight loss, increased firmness, and good looks	[91]
Broccoli, cauliflower, carrot, and chayote	Low methoxyl pectin + carnauba wax + glycerol + ascorbic acid	Conservation of sensory quality	[92]
Pear	Candelilla wax + Arabic gum + jojoba oil + pomegranate polyphenols	Increased shelf life, maintaining product quality	[93]
Mango	Cassava starch + citrus pectin	Shelf life extension	[94]
Strawberry	Tara gum + lipids (beeswax, shellac) + glycerol	Reduction of the rate of respiration, delay of the senescence process, and loss of texture	[95]
Guava	Whey protein concentrate + glycerol + oregano extract	Delay of the ripening process	[96]
Strawberry	Aloe vera + sodium alginate	Improvement in quality parameters (less weight loss, greater firmness, greater color retention, greater titratable acidity)	[97]
Tihuixocote	Tejocote pectin	Delay of the rate of pulp and skin loss of firmness	[98]

**Table 4:** Studies of the application of bioactive compounds on postharvest products for the inhibition of microorganisms.

	Bioactive compound/extraction source	Application technology	Inhibited microorganism	Microbiological technique used	Results obtained	Reference
Apple	Antioxidants, phenolic compounds, and flavonoids/residues (shell flour) of *Acca sellowiana* or “guava de Brazil”	Films	*Escherichia coli* *Salmonella typhimurium* *Pseudomonas aeruginosa*	Disc broadcast	The highest inhibition of the microorganisms tested was achieved in the films with the highest concentration of shell residues (3%).	[109]
In vitro	Polyphenols, flavonoids/broccoli leaves, cauliflower, and cabbage	ND	*Alternaria* spp.	Poison plaque	Cauliflower extracts presented the highest percentage of inhibition (24.14 ± 0.58%) compared to the rest of the compounds.	[110]
Orange	Polyphenols/pomegranate husk	Edible coatings	*Penicillium digitatum*	Inoculation of the fungus in the fruit with subsequent coating; diameters of the areas affected by the fungus were measured five days after inoculation	Films with aqueous extract of the pomegranate peel (0.361 g) were able to inhibit the fungus *Penicillium digitatum* that causes the green fungus in oranges.	[111]
Fresh cut mango	Gallic acid/mango residues	Immersion in aqueous extracts	Aerobic mesophilic bacteria, fungi, and yeasts	Plate count	80% inhibition of aerobic mesophiles 79% inhibition of fungi and yeasts.	[112]
In vitro	Phenolic acids, flavonoids/xoconostle (*Opuntia oligacantha*), orange essential oil, soy lecithin	Nanoemulsion with extracts of xoconostle, orange, and soy	*Colletotrichum gloeosporioides*	Well diffusion	The fungus was inhibited with the 4.15 mm nanoemulsion compared to the control, being the highest inhibition in the study.	[113]

## Data Availability

The data supporting this systematic review are from previously reported studies and datasets, which have been cited.
